# MicroRNA-214 promotes alveolarization in neonatal rat models of bronchopulmonary dysplasia via the PlGF-dependent STAT3 pathway

**DOI:** 10.1186/s10020-021-00374-4

**Published:** 2021-09-16

**Authors:** Zhi-Qun Zhang, Hui Hong, Jing Li, Xiao-Xia Li, Xian-Mei Huang

**Affiliations:** Department of Neonatology, Affiliated Hangzhou First People’s Hospital, Zhejiang University School of Medicine, No. 261, Huansha Road, Hangzhou, 310000 Zhejiang Province People’s Republic of China

**Keywords:** MicroRNA-214, PlGF, STAT3 pathway, Bronchopulmonary dysplasia, Alveolarization

## Abstract

**Background:**

Recently, the role of several microRNAs (miRNAs or miRs) in pulmonary diseases has been described. The molecular mechanisms by which miR-214 is possibly implicated in bronchopulmonary dysplasia (BPD) have not yet been addressed. Hence, this study aimed to investigate a putative role of miR-214 in alveolarization among preterm neonates with BPD.

**Methods:**

Microarray-based gene expression profiling data from BPD was employed to identify differentially expressed genes. A BPD neonatal rat model was induced by hyperoxia. Pulmonary epithelial cells were isolated from rats and exposed to hyperoxia to establish cell injury models. Gain- and loss-of-function experiments were performed in BPD neonatal rats and hyperoxic pulmonary epithelial cells. MiR-214 and PlGF expression in BPD neonatal rats, and eNOS, Bcl-2, c-myc, Survivin, α-SMA and E-cadherin expression in hyperoxic pulmonary epithelial cells were measured using RT-qPCR and Western blot analysis. The interaction between PlGF and miR-214 was identified using dual luciferase reporter gene and RIP assays. IL-1β, TNF-a, IL-6, ICAM-1 and Flt-1 expression in the rat models was measured using ELISA.

**Results:**

The lung tissues of neonatal rats with BPD showed decreased miR-214 expression with elevated PlGF expression. PlGF was found to be a target of miR-214, whereby miR-214 downregulated PlGF to inactivate the STAT3 pathway. miR-214 overexpression or PlGF silencing decreased the apoptosis of hyperoxic pulmonary epithelial cells in vitro and restored alveolarization in BPD neonatal rats.

**Conclusion:**

Overall, the results demonstrated that miR-214 could facilitate alveolarization in preterm neonates with BPD by suppressing the PlGF-dependent STAT3 pathway.

**Supplementary Information:**

The online version contains supplementary material available at 10.1186/s10020-021-00374-4.

## Background

Bronchopulmonary dysplasia (BPD), characterized by decreased lung growth, is a chronic lung disease that can affect preterm infants. It remains a major cause of neonatal morbidity and is often accompanied by serious adverse consequences (Isayama et al. 2017; Will et al. 2019). Approximately 45% of preterm infants whose gestation is less than 29 weeks are found to have BPD (Stoll et al. 2015). It has been documented that hyperoxia-induced acute lung injury is a key promoter of BPD pathogenesis in preterm infants (Syed et al. 2017). It is also well known that reduced lung alveolarization can cause the lungs to be incapable of effectively exchanging gas (Ruiz-Camp et al. 2019). However, the underlying molecular mechanisms are still largely unknown.

Through the mediation of cellular proliferation, differentiation and metastasis, microRNAs (miRNAs or miRs) are involved in the pathogenesis of multiple diseases and thus have great potential as therapeutic targets (Du et al. 2013). miRNAs have been also implicated in the pathogenesis of BPD due to their regulatory roles in alveolarization and vascularization (Shah et al. 2020; Das et al. 2021). miR-124 is shown to be downregulated during lung development in neonatal rats, and is mainly expressed in the epithelial cells during the late stage of the lung development (Wang et al. 2015a, b). In lung cancer, miR-214-3p has been demonstrated to downregulate fibroblast growth factor receptor 1, leading to beneficial effects in patients (Yang et al. 2019). Additionally, downregulation of miR-214 has been noted to reverse erlotinib resistance in non-small-cell lung cancer by upregulating its direct target gene LHX6 (Liao et al. 2017). In our study, placental growth factor (PlGF) was predicted to be the target gene of miR-214. Of note, PlGF has been considered as a potential biomarker for BPD occurrence (Zhang et al. 2014). Furthermore, PlGF has been found to increase under hyperoxic exposure, and downregulating PlGF can ameliorate hyperoxia-induced lung impairment in neonatal rats (Zhang et al. 2020). PlGF has also been noted to accelerate the phosphorylation of signal transducer and activator of transcription 3 (STAT3) in vascular smooth muscle cells exposed to hypoxia (Bellik et al. 2005). Moreover, neonatal exposure to hyperoxia reportedly leads to a significant increase in STAT3 mRNA expression in pulmonary endothelial cells (Chao et al. 2018). In this regard, we hypothesized that a regulatory network of the miR-214/PlGF/STAT3 pathway may be involved in BPD. Therefore, the current study aimed to verify an involvement of the miR-214/PlGF/STAT3 axis in BPD and elucidate its relevant molecular mechanisms.

## Materials and methods

### Compliance with ethical standards

Animal experiment protocols were approved by the Experimental Animal Ethics Committee of Affiliated Hangzhou First People’s Hospital, Zhejiang University School of Medicine (Approval Number: 2019-004-01). All animal experiments were performed in accordance with the Guide for the Care and Use of Laboratory Animals published by the US National Institutes of Health. Extensive efforts were made to ensure minimal suffering of the included animals during the study.

### Analysis of the BPD gene expression dataset and bioinformatics prediction

The gene expression dataset GSE25293 (Dong et al. 2012) of mouse BPD models was retrieved from the Gene Expression Omnibus (GEO) database (https://www.ncbi.nlm.nih.gov/gds). The GSE25293 dataset contains 4 normal mouse samples and 6 BPD mouse samples, with the mRNA annotation platform GPL1261 ([Mouse430_2] Affymetrix Mouse Genome 430 2.0 Array) and the miRNA annotation platform of GPL11199 (Applied Biosystems Mouse Taqman miRNA Expression Assays A and B). Differential analysis was conducted using the R package “limma” (http://www.bioconductor.org/packages/release/bioc/html/limma.html) to identify the significant highly expressed genes, with |logFC|> 1.0 and *p* value < 0.05 set as the threshold. The DIANA TOOLS (http://diana.imis.athena-innovation.gr/DianaTools/), miRWalk (energy < -30) (http://mirwalk.umm.uni-heidelberg.de), mirDIP (Integrated Score > 0.6) (http://ophid.utoronto.ca/mirDIP/), miRDB (http://www.mirdb.org) and miRSearch (https://www.exiqon.com/miRSearch) databases were used to analyze the intersected upstream miRNAs in the human body, and specific information of miRNA was obtained from the miRDB database (http://www.mirdb.org). A box plot was drawn using R language (version 3.6.3) to extract the key miRNA expression data from the miRNA dataset GSE25293 obtained using the platform GPL11199 in the GEO database. Protein–protein interaction (PPI) analysis was performed using the String website (https://string-db.org) to identify proteins that could potentially bind to BPD. Cytoscape (https://cytoscape.org) was used to plot the PPI network, and downstream regulatory pathways were predicted based on the existing literature.

### Establishment of hyperoxia-induced BPD neonatal rat models

Twelve specific-pathogen-free (SPF) pregnant Sprague–Dawley (SD) rats (gestational age of 15 days; Shanghai SLAC Laboratory Animal Co., Ltd., Shanghai, China) were raised at 22 ± 3 °C with 60 ± 5% humidity and 12 h light–dark cycle. Each pregnant rat was housed individually and was self-delivered after 1 week of acclimation. After delivery, 72 neonatal rats with the birth time 12 h apart were randomly grouped into the hyperoxia treatment group and the control group. The hyperoxia-treated rats with BPD were classified into the miR-214 negative control (NC) (BPD rats infected with adenoviral particles expressing NC for miR-214 agomir), miR-214 agomir (BPD rats infected with adenoviral particles expressing miR-214 agomir), miR-214 NC + PlGF vector (BPD rats infected with adenoviral particles expressing NC for miR-214 agomir and PlGF vector), and miR-214 agomir + PlGF (BPD rats infected with adenoviral particles expressing miR-214 agomir and PlGF) groups (n = 12 for each treatment). The oxygen concentration used in the hyperoxia group was 95%, and that used in the control group was 21%. The above conditions were continued until the end of the experiment. To prevent oxygen poisoning, the rats in the hyperoxia group were housed in a normal environment every 24 h. The neonatal pups were raised to 1 week in age and then injected with pAd-U6-MCS-CMV-GFP adenovirus vector. The recombinant adenovirus was propagated and amplified in HEK293 cells. The virus was injected via the tail vein at the volume of 30 μL at 1 × 10^8^ TU at a time interval of 3 days. miR-214 NC (5′-CGAUCGCAUCAGCAUCGAUUGC-3′), miR-214 agomir (5′-ACAGCAGGCACAGACAGGCAGU-3′), PlGF overexpression vector, and empty vector were purchased from RiboBio (Guangdong, China). The neonatal rats used for BPD modeling were placed in a plexiglas normobaric oxygen box with continuous oxygen input. In the box, the concentration of oxygen was maintained at 95%, and sodium lime absorbed CO_2_, with the temperature of 25–27 °C and the humidity of 50–70%. The control rats (air group) were exposed to air (at 21% oxygen concentration), and the remaining experimental conditions and operations were the same as those in the hyperoxia treatment group. The box was routinely opened for 30 min every day, water and food were added, and the litter was replaced. The postpartum rats were exchanged with the control group to avoid a decrease in the feeding ability of postpartum rats because of oxygen toxicity. The rats in the control group were placed in the same room, with similar experimental control factors to those in the hyperoxia treatment group. Three neonatal rats were randomly selected from the two groups by a random number generation method on the 3rd, 7th, and 14th days after the experiment began and then received an intraperitoneal injection of 90 mg/kg pentobarbital sodium for anesthesia. Next, the abdominal cavity was immediately opened, and the right lung was removed and placed in an RNase-free cryovial (Eppendorf, Hamburg, Germany). After rapid freezing with liquid nitrogen, the lungs were stored in a − 80 °C refrigerator for subsequent reverse transcription quantitative polymerase chain reaction (RT-qPCR) and Western blot analysis. Next, 40 g/L paraformaldehyde was slowly injected into the rats through the left bronchus until the apex of the lung was inflated. The lung was placed in an embedding box, and 40 g/L paraformaldehyde solution was added for overnight fixation and subsequent use.

### Enzyme-linked immunosorbent assay (ELISA)

Lung tissues of 3 rats in each group were collected on the 14th day of modeling, and 100 mg of frozen tissues were weighed and homogenized. After freezing and thawing, the homogenized tissues were sonicated for 10 min and incubated at 4 °C for 1 h. After centrifugation at 120,000×*g*, the supernatant was collected for measurement of interleukin (IL)-1β, tumor necrosis factor-α (TNF-ɑ) and IL-6 levels. Briefly, the strips used in the experiment were equilibrated at room temperature for 20 min. The standard and sample wells were set separately, and 50 μL of standards (IL-1β, TNF-ɑ and IL-6) were added at different concentrations. Then, the wells were supplemented with 10 μL of samples and then with 40 μL of sample dilution. The blank wells were not subjected to treatment. Next, 100 μL of the horseradish peroxidase (HRP)-labeled antibody to be detected was added to the standard wells and the sample wells, and the blank wells were not subjected to treatment. The wells were sealed with microplate sealers, followed by 60-min incubation at 37 °C. Thereafter, the liquid was discarded and the experimental strips were washed in a fully automatic washing machine. Then, 50 μL of substrates A and B were added to each well, followed by 15-min incubation at 37 °C in subdued light. Subsequently, 50 μL of stop buffer was added to each well, and the mixture was allowed to stand for 15 min, after which the optical density (OD) value of each well was measured at a wavelength of 450 nm.

### Histological analysis

Lung tissues of 3 rats in each group were collected on the 14th day of modeling, fixed with 4% paraformaldehyde for 24 h, sequentially dehydrated with 80%, 90% and 100% ethanol and n-butanol respectively, and immersed in a wax box at 60 °C. Following xylene dewaxing and hydration, the sections were first stained with hematoxylin (Solarbio, Beijing, China) for 2 min and then with eosin for 1 min, followed by gradient ethanol dehydration, xylene clearing, and neutral rubber fixing. Finally, the morphological changes in lung tissues were observed and analyzed under an optical microscope (XP-330, Bingyu Optical Instrument Co., Ltd., Shanghai, China).

### Bronchoalveolar lavage fluid (BALF) examination

Three rats from each group were anesthetized on the 14th day of modeling by intraperitoneal injection of 1% sodium pentobarbital solution at 80 mg/kg, and fixed on a simple operation table. The thoracic cavities of rats were exposed after being euthanized by exsanguination, after which the pleural tissues around the trachea were bluntly separated, the trachea was fully exposed, and a stab was made at the 1/3 of the trachea using a needle. The tip of the 18G indwelling needle had been previously trimmed into a hernia type, and tracheal intubation was performed along the trial point and ligated with a surgical line. Next, 1 mL of precooled sterile normal saline was used to perfuse the lung tissues of the rats three times, which were collected for subsequent use. The BALF was collected, stored in a precooled Eppendorf tube, and subsequently centrifuged at 290×*g* for 20 min at 4 °C. The supernatant was stored in a − 80 °C freezer for subsequent use. The cell precipitate was resuspended in 100 μL of phosphate buffer solution (PBS), after which 50 μL of the suspension was smeared, and stained with Swiss Giemsa. The number of neutrophils was counted under an oil immersion lens, and the total number of cells in 10 μL of the suspension was counted using a hemocytometer.

### Alveolar epithelial cell sorting using immunomagnetic beads

SPF SD rats (a gestational age of 15 days; Shanghai SLAC Laboratory Animal Co., Ltd., Shanghai, China) were raised at 22 ± 3 °C with humidity of 60 ± 5% and a 12-h the light–dark cycle. The pregnant mice were euthanized (Carbon dioxide asphyxiation: the animals were treated with 100% CO_2_ for 10 min in a clean container which was slowly bubbled with carbon dioxide at 5 L/min (16.7% volume displaced/min) under aseptic conditions. After euthanasia using carbon dioxide asphyxiation, the animals were examined one by one for thorough death (the animals were determined to be immobile and not breathing with dilated pupils. After CO_2_ was turned off, the animals were observed for an additional 2 min to confirm death.), and cervical dislocation was additionally conducted if the animals were found living. The fetal rats were removed by cesarean section from the SD rats and transected at the chest. Next, their lungs were removed and placed in precooled PBS to remove residual nonlung tissues. The lungs were perfused with 1 mL of dispase via the trachea, and the trachea was tied off with a string. The lungs were treated in 2 mL of dispase for 30 min at 37 °C, followed by mincing. The suspension was sequentially filtered by through 70-, 40-, and 10-µm nylon meshes, followed by centrifugation at 200×*g* for 10 min. The pellet was resuspended in Dulbecco’s modified Eagle’s medium (DMEM; Invitrogen, Karlsruhe, Germany). CD14 magnetic beads (130–050-201, Miltenyi Biotech, Bergisch-Gladbach, Germany) were added to the cells (20 μL/10^7^ cells) and mixed well, and the cells were cultured for 15 min at 4–8 °C. The cells were then washed with buffer solution (1 mL/10^7^ cells) and centrifuged at 453×*g* for 10 min, and the supernatant was completely removed. The cells were resuspended in 500 μL of buffer solution (pH 7.2; PBS supplemented with 0.5% bovine serum albumin and 2 mM minocycline ethylenediaminetetraacetic acid), and the cell suspension was added to a MS separation column (130042201; Miltenyi, Bergisch Gladbach, Germany). The unlabeled cells that had flowed out first were collected, and were the negative cells. The MS separation column was washed with 1500 μL of buffer solution. The separation column was removed from the magnetic field, and the cells retained on the column were quickly eluted with 1 mL of buffer solution. These cells were magnetically labeled positive cells. Under a modified Barthel microscope, additional dark particles were observed in the cytoplasm. The characteristic osmiophilic bodies, including lamellar bodies and cell membranes, were observed to have distinct microvilli under with transmission electron microscopy (TEM). This indicated that type II alveolar epithelial cells in the fetal rats were successfully isolated.

### Development of hyperoxia-induced cell injury models

After 2 days of growth, the cells were seeded into a 6-well plate at a density of 3 × 10^5^ cells/well and conventionally cultured until 75% cell confluence. Pulmonary epithelial cells were randomly assigned into the control (cells exposed to air for 24 h), hyperoxia (cells exposed to hyperoxia for 24 h), miR-214 NC (cells infected with adenoviral particles expressing NC for miR-214 mimic before cell exposure to hyperoxia for 24 h), miR-214 mimic (cells infected with adenoviral particles expressing miR-214 mimic before cell exposure to hyperoxia for 24 h), miR-214 NC + PlGF vector (cells infected with adenoviral particles expressing NC for miR-214 mimic and PlGF vector before cell exposure to hyperoxia for 24 h), miR-214 NC + PlGF (cells infected with adenoviral particles expressing NC miR-214 mimic and PlGF before cell exposure to hyperoxia for 24 h), and miR-214 mimic + PlGF (cells infected with adenoviral particles expressing NC for miR-214 mimic and PlGF vector before cell exposure to hyperoxia for 24 h) groups. The cells in the air-exposed and hyperoxia-exposed groups were placed in closed oxygen chambers with oxygen volume fractions of 21% and 85%, respectively. The pulmonary epithelial cells were infected with adenovirus and placed in a closed oxygen chamber with 85% oxygen volume fraction, for 24 h. The sequences of miR-214 mimic and miR-214 NC used in vitro were identical to those of miR-214 agomir and miR-214 NC used in vivo.

### TEM

After the pulmonary epithelial cells were centrifuged at 12,882×*g* for 10 min, the supernatant was discarded. The cells were fixed with 4% glutaraldehyde at 4 °C for more than 2 h and then with 1% osmium tetroxide for 2 h, and dehydrated with gradient ethanol and acetone. The cells were next immersed in epoxy resin, embedded, polymerized, and then prepared into semithin sections with a thickness of 0.5 μm. The sections were positioned under a microscope, stained with uranyl acetate and lead citrate, observed, and photographed under a TEM (H-7500).

### RNA binding protein immunoprecipitation (RIP)

The pulmonary epithelial cells were washed with 10 mL of cool PBS two times, and then 10 mL of PBS was added. The pulmonary epithelial cells were scraped off with a cell scraper and transferred into a centrifuge tube. They were centrifuged at 200×*g* for 5 min at 4 °C, RIP lysis buffer was added, and the cells were mechanically dissociated, mixed thoroughly, and lysed on ice for 5 min to prepare cell lysates. Next, 50 μL of magnetic beads were added into each tube and mixed well, after which 0.5 mL of RIP wash buffer was added to rinse the magnetic beads, and 100 μL of RIP wash buffer was added to resuspend beads. Then, 5 μg of Ago2 antibody was added to the tubes and incubated while rotating for 30 min at room temperature. The supernatant was discarded, and the beads were washed twice with 0.5 mL of RIP wash buffer for subsequent experiments. Next, 900 μL of RIP immunoprecipitation buffer was added to the magnetic bead-antibody mixture, after which the centrifugation was carried out at 16,000×*g* for 10 min at 4 °C. The supernatant was collected and transferred into an Eppendorf tube, and then 100 μL of supernatant was transferred to the tube containing the magnetic bead-antibody with 1.0 mL as the final volume of the immunoprecipitation reaction. Incubation was conducted overnight at 4 °C. Next, the magnetic beads were washed 6 times with 0.5 mL of RIP wash buffer, and the RNA was purified with 150 μL of proteinase K buffer for 30 min at 55 °C. The RNA was extracted by a conventional TRIzol method followed by RT-qPCR detection.

### Dual luciferase reporter gene assay

The artificially synthesized PlGF 3' untranslated region (UTR) gene fragment was constructed into a pMIR reporter (Promega, Madison, WI, USA). A complementary sequence with mutation (MUT) of the seed sequence was designed based on the wild type (WT) of PlGF and constructed into the pMIR-reporter reporter plasmid. The correctly sequenced luciferase reporter plasmids WT and MUT were co-transfected with miR-214 mimic and miR-214 NC into 293T cells (Hunan Fenghui Biological Co., Ltd., Hunan, China) at the second passage. Forty-eight hours after transfection, the cells were collected and lysed, and luciferase activity was measured using the Dual-Luciferase Reporter Assay System (Promega, Madison, WI, USA).

### RT-qPCR assay

Total RNA was extracted from tissues of 3 rats in each group on days 1, 3, 7, and 14 and from cells using the TRIzol reagent (Invitrogen Inc., Carlsbad, CA, USA) and reverse transcribed into complementary DNA (cDNA) using miRNA First Strand cDNA Synthesis (Tailing Reaction) (B532451, Shanghai Sangon Biotechnology Co. Ltd., Shanghai, China) and MightyScript First Strand cDNA Synthesis Master Mix (B639251, Sangon). The reverse transcribed cDNA was diluted to 10 times. The expression of relevant genes was analyzed and normalized to U6 (for miRNA), and glyceraldehyde-3-phosphate dehydrogenase (GAPDH) (for other genes). Fold changes were calculated using relative quantification (the 2^−∆∆Ct^ method): ∆∆Ct = ∆Ct model group − ∆Ct normal group, ∆Ct = Ct (target gene) − Ct (internal reference). The primer sequences used are shown in Additional file 1: Table S1.

### Western blot analysis

The lung tissues of 3 rats in each group on days 1, 3, 7, and 14 or cells were added to lysis buffer, shaken on a vortex agitator, and centrifuged at 28,985×*g* for 30 min at 4 °C to remove tissues or cell debris. The supernatant was collected, and the total protein concentration was measured using a bicinchoninic acid (BCA) kit. Fifty micrograms of protein was subjected to 10% sodium dodecyl sulfate polyacrylamide gel electrophoresis and electroblotted to polyvinylidene fluoride membranes by the wet transfer method. After being blocked with 5% skim milk powder at room temperature for 1 h, the membrane was then probed with diluted primary antibodies from Cell Signaling Technologies (Danvers, MA, USA) against Survivin (#2808S), GAPDH (#5174), B-cell lymphoma-2 (Bcl-2, #3498) and c-myc (#13987), cleaved caspase-3 (#9661) and primary antibodies from Abcam (Cambridge, UK) against Bax (ab32503), PlGF (ab256453), E-cadherin (ab11512) and α-smooth muscle actin (α-SMA, ab32575), and then diluted, based on the instruction provided by the manufacturers. The membrane was re-probed with HRP-labeled secondary antibody for 1 h. The membrane was placed on a clean glass plate. The immunocomplexes on the membrane were visualized using an enhanced chemiluminescence (ECL) fluorescence detection kit (BB-3501, Amersham, Little Chalfont, UK), and band intensities were quantified using a Bio-Rad image analysis system with Quantity One v4.6.2 software. The ratio of the gray value of the target band to GAPDH was considered representative of the relative protein expression.

### Statistical analysis

Data analyses were conducted using SPSS 21.0 (IBM Corp, Armonk, NY, USA). Measurement data were summarized as mean ± standard deviation. Unpaired *t*-test was conducted to compare the data with a normal distribution and homogeneity of variance between two groups. Data comparisons between multiple groups were performed using one-way analysis of variance (ANOVA), followed by a Tukey’s multiple comparisons post hoc test. Data comparisons at different time points were performed by two-way ANOVA, followed by a Bonferroni post hoc test for multiple comparisons. A value of *p* < 0.05 was considered statistically significant.

## Results

### miR-214 is predicted to orchestrate PlGF expression to mediate the STAT3 pathway in BPD

**Fig. 1:**
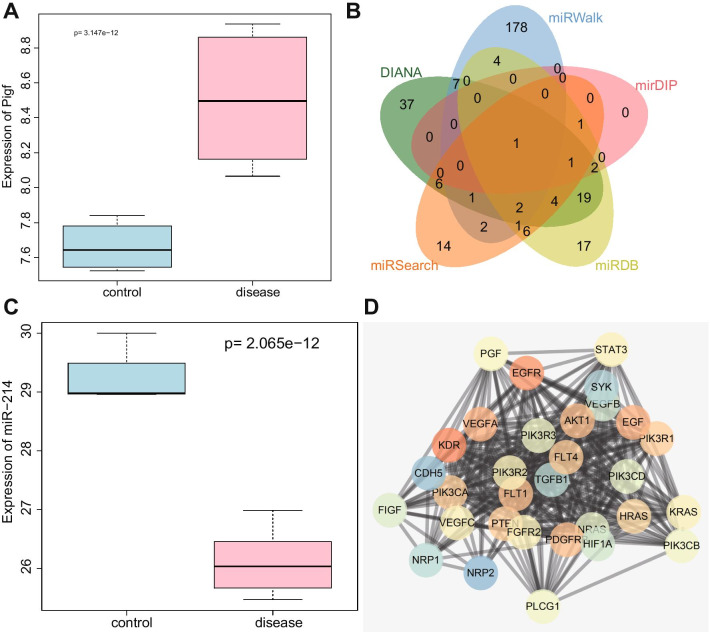
The PlGF/miR-214/STAT3 axis is predicted to be involved in BPD. **A** PlGF expression in the GSE25293-GPL1261 dataset; the blue box on the left indicates the expression level in normal samples, and the red box on the right indicates the expression level in BPD samples. **B** The Venn diagram depicting the intersection of upstream miRNAs of PlGF predicted by the DIANA Tools, miRWalk, mirDIP, miRDB, and miRSearch databases. **C** A box plot depicting miR-214 expression in the GSE25293-GPL11199 dataset; the blue box on the left indicates the expression in normal samples, and the red box on the right indicates the expression in BPD samples. **D** PPI network analysis of PlGF (PGF); darker hue of the red gene sphere indicates greater importance and darker hue of blue gene sphere indicates lower importance

It has been reported that PlGF is an important gene participating in BPD in preterm infants, but the regulatory pathways of this gene are still unknown, representing an area of significant research potential (Janer et al. 2008). Therefore, we explored the modulatory mechanisms of PlGF in BPD. The boxplot of PlGF expression from GSE25293-GPL1261 (Fig. 1A) illustrated that PlGF was highly expressed in BPD. The predicted results from the DIANA, miRWalk, mirDIP, miRDB and miRSearch databases revealed that the number of miRNA upstream of PlGF (actually PGF was used during prediction) were 80, 196, 5, 58, and 39, respectively. Only one intersecting miRNA, miR-214, was obtained, as shown by the Venn diagram (Fig. 1B). In addition, the boxplot of miR-214 expression depicted that miR-214 was downregulated in neonatal rats with BPD induced by hyperoxia (Fig. 1C). A previous study has shown that miR-214-3p is downregulated in lung tumor tissues and represses lung cancer cell proliferation (Chen et al. 2018). PPI analysis indicated that PlGF (PGF) may be related to several pathways (Fig. 1D), and studies have shown that high expression of PlGF can activate STAT3 pathway (Rovani et al. 2017; Qi et al. 2018). Based on these data, we hypothesized that miR-214 could regulate the expression of PlGF and further regulate the STAT3 pathway, thus affecting the progression of BPD in preterm infants.

### miR-214 is downregulated but PlGF is upregulated in the lung tissues of hyperoxia-induced BPD neonatal rats

**Fig. 2:**
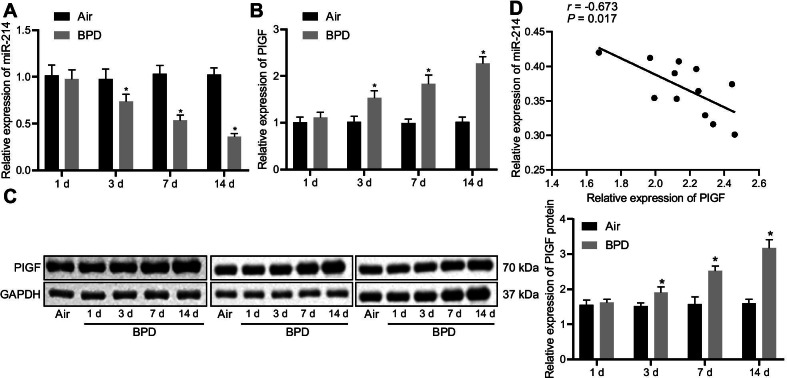
Decreased miR-214 expression and increased PlGF expression in the lung tissues of neonatal rats with BPD. **A** miR-214 expression in lung tissues of neonatal rats with BPD as determined by RT-qPCR and normalized to U6. **B** mRNA level of PlGF in lung tissues of neonatal rats with BPD as determined by RT-qPCR and normalized to GAPDH. **C** Western blot analysis of PlGF protein expression in lung tissues of neonatal rats with BPD normalized to GAPDH. **D** Correlation analysis of miR-214 and PlGF expression in lung tissues of neonatal rats on the 14th day. The data are summarized as mean ± standard deviation. *p < 0.05 vs. neonatal rats exposed to air. Data comparisons at different time points were performed using two-way ANOVA, followed by a Bonferroni or Tukey’s post hoc test for multiple comparisons. n = 3 for rats in each group

To verify the above hypothesis, a hyperoxia-induced neonatal rat model with BPD was established. RT-qPCR data showed that on the 3rd, 7th, and 14th days, miR-214 expression in lung tissues was lower in neonatal rats with BPD than in air-treated neonatal rats (Fig. 2A). The results of RT-qPCR and Western blot analysis displayed that PlGF expression was elevated in the lung tissues of neonatal rats with BPD as compared with air-treated neonatal rats on the 3rd, 7th, and 14th day in a time-dependent manner (Fig. 2B, C). Correlation analysis manifested a negative correlation between miR-214 and PlGF expression in the tissues of BPD neonatal rats on the 14th day (Fig. 2D). These results implied that miR-214 expression was decreased and that PlGF was elevated in the lung tissue of neonatal rats exposed to hyperoxia with a negative correlation between them.

### Overexpression of miR-214 restores alveolarization in neonatal rats with BPD in vivo

**Fig. 3:**
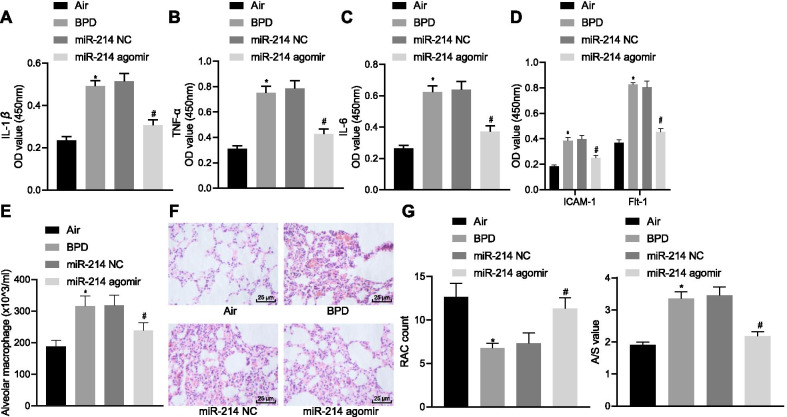
miR-214 overexpression induces alveolarization in neonatal rats with BPD. BPD neonatal rats were injected with miR-214 NC and miR-214 agomir. **A** ELISA quantification of the proinflammatory factor IL-1β in rats. **B** ELISA quantification of the proinflammatory factor TNF-ɑ in rats. **C** ELISA quantification of the proinflammatory factor IL-6 in rats. **D** ELISA quantification of ICAM-1 and Flt-1 levels in rats. **E** Giemsa staining of the number of macrophages in rats. **F** H&E staining of pulmonary microvascular (× 400). **G** H&E staining showing the number of alveoli, and alveolar growth. Data are summarized as mean ± standard deviation. *p < 0.05 vs. neonatal rats exposed to air. ^#^*p* < 0.05 vs. BPD neonatal rats treated with miR-214 NC. Data comparisons between multiple groups were performed using one-way ANOVA and Tukey's for post hoc test. n = 3 for rats in each group

To validate the effect of miR-214 overexpression on neonatal rats with BPD, hyperoxia-induced neonatal rats with BPD were injected with miR-214 NC and miR-214 agomir. After injection of miR-214 agomir, miR-214 expression was obviously elevated in BPD rats (Additional file 2: Fig. S1A). Compared with neonatal rats exposed to air, BPD neonatal rats exhibited increased levels of inflammatory factors (IL-1β, TNF-ɑ, IL-6, Flt-1 and ICAM-1) (Fig. 3A–D) and an increased number of macrophages (Fig. 3E, Additional file 3: Fig. S2A), and these trends were reversed by miR-214 agomir treatment. Hematoxylin–eosin (H&E) staining data revealed that compared with neonatal rats exposed to air, BPD neonatal rats exhibited a reduction in the number of alveoli and a simplified structure. Moreover, the alveolar wall ruptured and merged into pulmonary bullae, and the ratio of alveolar area/pulmonary septal area was increased. After miR-214 agomir treatment, BPD neonatal rats exhibited an increased number of alveoli with mature lung tissue structure, regular alveolar structure, and reduced septal area, accompanied by a reduction in the ratio of alveolar area/pulmonary septal area (Fig. 3F, G). These results indicated that the overexpression of miR-214 could promote alveolarization in neonatal rats with BPD.

### Overexpression of miR-214 decreases pulmonary epithelial cell apoptosis in vitro

**Fig. 4:**
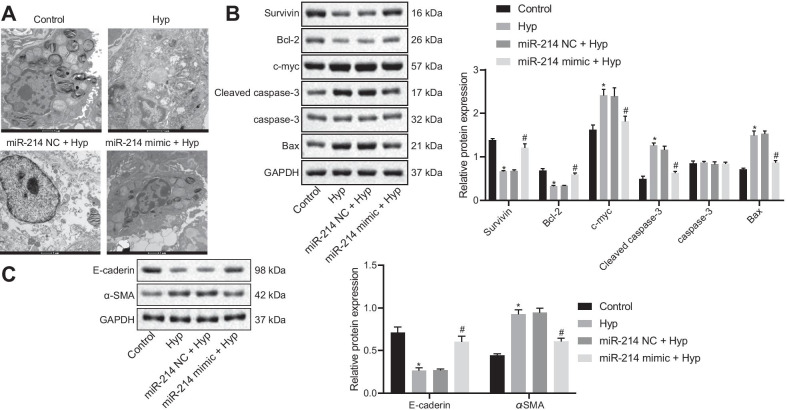
miR-214 overexpression suppresses apoptosis and fibrosis of embryonic pulmonary epithelial cells of rats with BPD. Hyperoxic pulmonary epithelial cells were transfected with miR-214 NC or miR-214 mimic. **A** The ultrastructure of alveolar epithelial cells under TEM (× 10,000). **B** Western blot analysis for antiapoptotic proteins Survivin and Bcl-2 and the proapoptotic proteins Bax, c-myc, and cleaved caspase-3 in embryonic pulmonary epithelial cells. **C** Western blot analysis for epithelial cell marker E-cadherin and the fibrosis marker α-SMA in embryonic pulmonary epithelial cells. The data are summarized as mean ± standard deviation. *p < 0.05 vs. pulmonary epithelial cells exposed to air. ^#^*p* < 0.05 vs. hyperoxic pulmonary epithelial cells treated with miR-214 NC. Multiple comparisons were performed using one-way ANOVA, followed by Tukey's post hoc test for multiple comparisons. Each experiment was repeated three times

To elucidate the effect of miR-214 on pulmonary epithelial cells, pulmonary epithelial cells were obtained from rats and transfected with miR-214 NC or miR-214 mimic. It was observed that miR-214 expression was significantly enhanced in miR-214 mimic-transfected pulmonary epithelial cells (Additional file 2: Fig. S1B). As observed with TEM, in contrast to the pulmonary epithelial cells exposed to air, the cytoplasmic lamellar structure of pulmonary epithelial cells treated with hyperoxia was destroyed, evidenced by the formation of relatively large vacuoles and an increase in the gap between blood-air barriers, and these effects could be rescued by miR-214 mimic transfection (Fig. 4A). The dysregulation of c-myc expression is the major cause of several types of cell apoptosis and further, the speed of cell apoptosis and its sensitivity to inducing factors depend on the content of cellular Myc protein. E-cadherin is a member of the Ca^2+^-dependent cell adhesion molecule family of adhesion molecules that effectively maintains normal epithelial cell morphology and structural integrity, whereas α-SMA, a marker of fibroblast activation and differentiation, is considered as an important factor in the process of organ fibrosis. The Western blot analysis results depicted in Fig. 4B demonstrated that in hyperoxic pulmonary epithelial cells, the levels of antiapoptotic proteins (Survivin and Bcl-2) were decreased, and the levels of proapoptotic proteins (Bax, cleaved caspase-3, and c-myc) were increased. miR-214 mimic treatment induced the opposite trends. Expression of the fibrosis marker α-SMA expression was increased, while expression of the epithelial cell marker E-cadherin expression was reduced in hyperoxic pulmonary epithelial cells, as compared with control cells, and this effect was blocked by the miR-214 mimic (Fig. 4C). Taken together, these data demonstrated that the overexpression of miR-214 could attenuate pulmonary epithelial cell alteration in BPD rats.

### PlGF is a target gene of miR-214

**Fig. 5:**
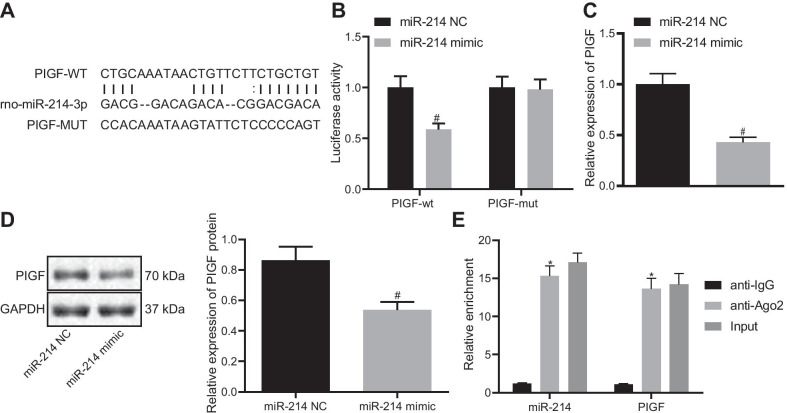
PlGF is identified as a target of miR-214. **A** The predicted binding sites of miR-214 and PlGF. **B** Dual luciferase reporter assay analysis of the binding of miR-214 to PlGF. **C** RT-qPCR determination of the mRNA level of PlGF following miR-214 overexpression in cells. **D** Western blot analysis of PlGF following miR-214 overexpression in cells. **E** RIP detection of the binding percentage of miR-214 and PlGF to Ago2, normalized to IgG binding. Data are summarized as mean ± standard deviation. *p < 0.05 vs. anti-IgG. ^#^*p* < 0.05 vs. cells transfected with miR-214 NC. An independent sample *t*-test was used for comparisons between two groups. Multiple group comparisons were performed using one-way ANOVA, followed by Tukey's post hoc test. Each experiment was repeated three times

Next, the regulatory mechanism of miR-214 was explored. Bioinformatics analysis was implemented using the web-based software RNA22 (https://cm.jefferson.edu/rna22/Precomputed/), which revealed binding sites existed between rno-miR-214 and PlGF (Fig. 5A). A dual luciferase reporter assay verified that co-transfection of the miR-214 mimic with the 3′-UTR of WT-PlGF reduced luciferase activity, whereas the 3′-UTR of MUT-PlGF showed no significant difference in luciferase activity (Fig. 5B), indicating that miR-214 could specifically bind to the 3′-UTR of PlGF and regulate its expression. In addition, RT-qPCR and Western blot analysis results depicted that the mRNA and protein levels of PlGF were reduced in cells treated with the miR-214 mimic (Fig. 5C, D). RIP experiments further validated that the enrichment of miR-214 and PlGF was higher in the Ago2 group than in the IgG group (Fig. 5E). Collectively, miR-214 targeted PlGF and regulated its expression.

### Overexpressed miR-214 promotes alveolarization by inactivating the PlGF-dependent STAT3 pathway in neonatal rats with BPD

**Fig. 6:**
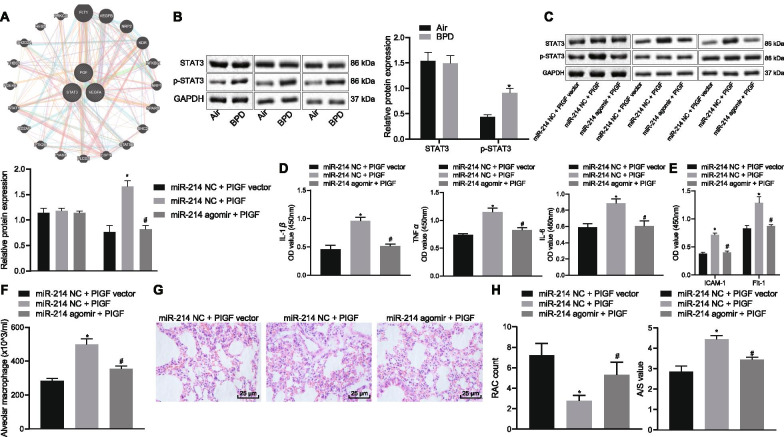
miR-214 overexpression blocks the effect of the activated STAT3 pathway on alveolarization by inhibiting PlGF in neonatal rats with BPD. **A** The KEGG and GeneMANIA databases were used to predict the regulatory pathways downstream of PlGF. **B** Western blot analysis of the activation of the STAT3 pathway in BPD neonatal rats normalized to GAPDH. Neonatal rats with BPD were then treated with miR-214 NC + PlGF vector, miR-214 NC + PlGF, or miR-214 agomir + PlGF. **C** Western blot analysis of the expression of STAT3 and phosphorylated STAT3 in lung tissues of BPD neonatal rats, normalized to GAPDH. **D** Quantification of the levels of the inflammatory factors IL-1β, TNF-ɑ, and IL-6 by ELISA. **E** Quantification of the levels of ICAM-1 and Flt-1 by ELISA. **F** Giemsa staining of the number of macrophages. **G** H&E staining of the formation of pulmonary alveoli (× 400). **H** H&E staining of the number of alveoli and alveolar growth. Measurement data are summarized as the mean ± standard deviation. *p < 0.05 vs. BPD rats treated with miR-214 NC + PlGF vector. ^#^*p* < 0.05 vs. BPD rats treated with miR-214 NC + PlGF. An independent sample *t*-test was used for comparisons between two groups. Comparisons between multiple groups were performed using one-way ANOVA, followed by Tukey's post hoc test for multiple comparisons. n = 3 for rats in each group

The downstream regulatory pathway of PlGF was predicted using the KEGG and GeneMANIA databases, and the results demonstrated that PlGF was closely related to the STAT3 pathway (Fig. 6A). Western blot analysis showed that STAT3 phosphorylation was increased in the lung tissue of neonatal rats exposed to hyperoxia (Fig. 6B). Neonatal rats with BPD were then treated with miR-214 NC + PlGF vector, miR-214 NC + PlGF, or miR-214 agomir + PlGF. The results documented that in the presence of miR-214 NC, PlGF treatment caused remarkable increase of PlGF mRNA expression, and in the presence of PlGF, miR-214 agomir resulted in reduction of PlGF mRNA expression (Additional file 2: Fig. S1C, D). PlGF overexpression elevated the expression of phosphorylated STAT3/STAT3 in lung tissues (Fig. 6C), the levels of IL-1β, TNF-ɑ, IL-6, ICAM-1 and Flt-1 (Fig. 6D–E), and the number of macrophages (Fig. 6F, Additional file 3: Fig. S2B), which was abrogated by treatment with miR-214 agomir + PlGF. Moreover, PlGF upregulation reduced the number of alveoli and led to an irregular structure. The alveolar wall ruptured to form a large pulmonary vesicle with an elevated alveolar area/pulmonary septal area ratio. Conversely, we observed an increased alveoli number, regular alveolar structures, and reduced septal area and ratio of alveolar area/pulmonary septal area in BPD rats treated with miR-214 agomir + PlGF (*p* < 0.05; Fig. 6G, H). These results suggested that miR-214 targeted PlGF to inhibit the STAT3 pathway, thus stimulating alveolarization in neonates with BPD.

### miR-214 overexpression reduces pulmonary epithelial cell apoptosis and fibrosis via inactivation of the PlGF-dependent STAT3 pathway in vitro

**Fig. 7:**
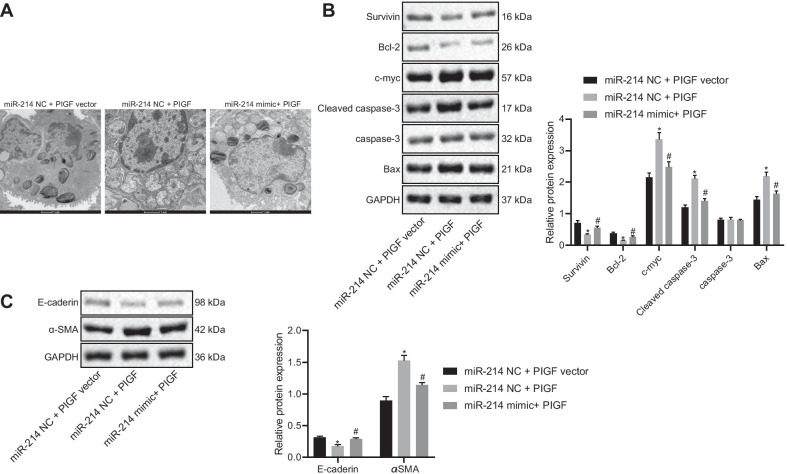
miR-214 overexpression blocks the effect of the activated STAT3 pathway on bronchial embryonic pulmonary epithelial cells by inhibiting PlGF. **A** The ultrastructure of alveolar epithelial cells under TEM (× 10,000). **B** Western blot analysis to quantify the expression of antiapoptotic proteins (Survivin and Bcl-2) and proapoptotic proteins (Bax, c-myc, and cleaved caspase-3) proteins in embryonic pulmonary epithelial cells. **C** Western blot analysis to quantify the expression of the epithelial cell marker E-cadherin and the fibrosis marker α-SMA in embryonic pulmonary epithelial cells. Data are summarized as mean ± standard deviation. *p < 0.05 vs. pulmonary epithelial cells transfected with miR-214 NC and PlGF NC. ^#^*p* < 0.05 vs. pulmonary epithelial cells transfected with miR-214 NC and PlGF. Multiple comparisons were performed using one-way ANOVA, followed by Tukey's post hoc test. Each experiment was repeated three times

To study the effect of miR-214 on the pulmonary epithelial cells of rats with BPD, pulmonary epithelial cells were obtained from rats and transfected with miR-214 NC + PlGF vector, miR-214 NC + PlGF, or miR-214 mimic + PlGF. PlGF mRNA expression was higher in pulmonary epithelial cells transfected with miR-214 NC + PlGF than in those transfected with miR-214 NC + PlGF vector, but was lower in pulmonary epithelial cells transfected with miR-214 mimic + PlGF than in those transfected with miR-214 NC + PlGF (Additional file 2: Fig. S1E, F). Under TEM, in the miR-214 NC + PlGF-treated rats, the cytoplasmic lamellar structure of the alveolar epithelium was destroyed, large vacuoles were formed, and the blood-air barrier gap was increased, as compared to miR-214 NC + PlGF vector-treated rats. The opposite results were observed in rats treated with miR-214 mimic + PlGF in comparison with miR-214 NC + PlGF-treated rats (Fig. 7A). Moreover, in comparison to transfection with miR-214 NC + PlGF vector, the expression of antiapoptotic proteins (Survivin and Bcl-2) and the fibrosis marker α-SMA was lower, whereas the expression of proapoptotic proteins (c-myc, Bax, cleaved caspase-3) and the epithelial cell marker E-cadherin was higher with miR-214 NC + PlGF treatment. The effect of miR-214 NC + PlGF transfection was negated by transfection with miR-214 mimic + PlGF (Fig. 7B, C). These results indicated that overexpression of miR-214 targeted PlGF to disrupt the STAT3 pathway, thereby restraining rat bronchial embryonic pulmonary epithelial cell apoptosis and fibrosis.

## Discussion

BPD, a respiratory condition that occurs in preterm neonates often leading to chronic respiratory compromise, is driven by several prenatal and/or postnatal factors (Principi et al. 2018). The known risk factors associated with BPD development in preterm neonates include low gestational age, preeclampsia, chorioamnionitis, and infiltration of the chorioamnion by neutrophils (Ogawa et al. 2017; Dravet-Gounot et al. 2018). The most likely underlying pathogenesis is constant inflammation in the lung; thus, corticosteroids, which exert a strong anti-inflammatory effect, have been employed in the treatment of BPD (Doyle et al. 2017). The present study was designed to explore a novel potential therapeutic target for promoting alveolarization in neonatal infants with BPD (a schematic diagram of the timeline of the experiments is represented in Additional file 4: Fig. S3). The in vitro and in vivo experiments demonstrated that miR-214 could induce alveolarization in neonates with BPD and suppress pulmonary epithelial cell apoptosis in vitro via PlGF-dependent STAT3 pathway blockade.

Our findings illustrated that miR-214 was downregulated in hyperoxia-induced BPD neonatal rats. miR-214, a miRNA precursor, plays a pivotal role in the pathogenesis of multiple human disorders, including cardiovascular diseases and cancers (Zhao et al. 2017; Sun et al. 2018). TWIST1-induced increase in the expression of miR-214 is shown to promote epithelial-to-mesenchymal transition and metastasis in lung adenocarcinoma (Liu et al. 2018). Additionally, our study showed that PlGF, which was highly expressed in the lung tissues of preterm rats with BPD, was negatively targeted by miR-214 and activated the STAT3 pathway. Consistently, PlGF was earlier reported to be highly expressed in BPD rats (Yang et al. 2015). An inverse correlation between miR-214 and PlGF was detected in our study. It is known that post-transcriptional miR-214 possesses the ability to modulate the expression of PlGF in lung tissues (Gonsalves et al. 2015). In addition, PlGF is also shown to increase the phosphorylation of STAT3 (Bellik et al. 2005). Notably, miR-214 has been demonstrated to downregulate the expression of STAT3 in human cervical and colorectal cancer cells (Chandrasekaran et al. 2017).

An important finding of our study was that miR-214 overexpression could downregulate IL-1β, TNF-α and IL-6, and thereby, promote alveolarization in hyperoxia-induced BPD neonatal rats. A previous study has indicated that adeno-associated 9-mediated restoration of miR-29b improves lung alveolarization in prematurely born infants with BPD exposed to hyperoxia (Durrani-Kolarik et al. 2017). The present study is the first to reveal the promotive effect of miR-214 on alveolarization in context of BPD, reflecting its role in regulating the progression of BPD. IL-1β is one of the key mediators of inflammation and is implicated in numerous diseases (Sitia et al. 2018). Considering the primary pathological features of BPD, IL-1β is found to contribute to excessive alveolar elastogenesis through its interaction with αvβ6, which serves as an epithelial or mesenchymal signaling molecule (Wang et al. 2015a, b). TNF is a ligand strongly linked with systemic inflammation in the human body (Cheng et al. 2014). TNF-ɑ overexpression is known not only to increase the release of glutamate but also decrease the cell cycling activity of marrow mesenchymal stem cells (Yue et al. 2019). IL-6 is a typical inflammatory cytokine that plays a functional role in a number of physiological inflammatory and immunological processes (Ataie-Kachoie et al. 2014). A strong correlation has been found between the dysregulation of IL-6 and moderate and severe BPD in preterm infants with low gestational age (Rocha et al. 2012). Of note, the elevation of miR-124 has been shown to significantly relieve the hyperoxia-triggered release of proinflammatory cytokines in pulmonary epithelial cells (Li et al. 2018). In general, PlGF overexpression is known to contribute to an exaggerated inflammatory state (Newell et al. 2017). Here, the experimental data demonstrated that miR-214 overexpression could reduce the expression of proinflammatory factors, thus enhancing alveolarization and inhibiting cell apoptosis of the lung epithelium in neonatal rats with BPD.

## Conclusions

**Fig. 8:**
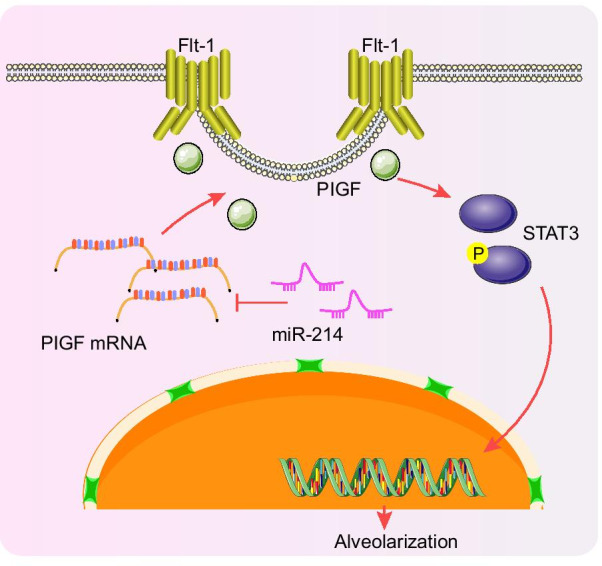
A schematic diagram illustrating the roles of the miR-214-PlGF-STAT3 regulatory network in preterm infants with BPD. miR-214 can inhibit the activation of the STAT3 pathway by inhibiting the transcription of its downstream target gene PlGF, ultimately enhancing alveolarization in preterm infants with BPD

In conclusion, the present study depicted that upregulated miR-214 can potentially block the activation of the STAT3 pathway by inhibiting its downstream target gene PlGF, ultimately promoting alveolarization in neonatal infants with BPD (Fig. 8). The discovery of miR-214 as a new regulator that controls the PlGF-STAT3 axis in BPD offers a fresh molecular insight that might be utilized in new therapy development for BPD. However, comprehensive molecular mechanisms implicated in the role of miR-214-PlGF-STAT3 pathway in BPD warrant further elucidation through pre-clinical and clinical evidence.

## Supplementary Information

**Additional file 1: Table S1.** Primer sequences of RT-qPCR

**Additional file 2: Fig. S1.** Validation of transfection efficiency in vivo and in vitro. A, RT-qPCR determination of miR-214 expression level in lung tissues of rats treated with miR-214 NC or miR-214 agomir. *p < 0.05 vs. the rats treated with miR-214 NC. B, RT-qPCR determination of miR-214 expression level in pulmonary epithelial cells transfected with miR-214 NC or miR-214 mimic. *p < 0.05 vs. pulmonary epithelial cells treated with miR-214 NC. C, RT-qPCR determination of PlGF mRNA expression in lung tissues of rats treated with miR-214 NC + PlGF vector, miR-214 NC + PlGF, or miR-214 agomir + PlGF. * p < 0.05 vs. BPD rats treated with miR-214 NC + PlGF vector. # p < 0.05 vs. BPD rats treated with miR-214 NC + PlGF. D, RT-qPCR determination of PlGF mRNA expression in lung tissues of BPD rats treated with PlGF vector or PlGF. * p < 0.05 vs. BPD rats treated with PlGF vector. E, RT-qPCR determination of PlGF mRNA expression in pulmonary epithelial cells transfected with miR-214 NC + PlGF vector, miR-214 NC + PlGF, or miR-214 mimic + PlGF. * p < 0.05 vs. pulmonary epithelial cells transfected with miR-214 NC + PlGF vector. # p < 0.05 vs. pulmonary epithelial cells transfected with miR-214 NC + PlGF. F, RT-qPCR determination of PlGF mRNA expression in pulmonary epithelial cells transfected with PlGF vector or PlGF. * p < 0.05 vs. pulmonary epithelial cells transfected with PlGF vector. Data are summarized as mean ± standard deviation. An independent sample t-test was used for comparisons between two groups. One-way ANOVA, followed by Tukey's post hoc test was used for multiple comparisons. Each experiment was repeated three times.

**Additional file 3: Fig. S2.** Representative images of Giemsa staining. A, Representative images of Giemsa staining of BPD neonatal rats injected with miR-214 NC and miR-214 agomir (× 400). B, Representative images of Giemsa staining of BPD neonatal rats treated with miR-214 NC + PlGF vector, miR-214 NC + PlGF, or miR-214 agomir + PlGF (× 400). 

**Additional file 4: Fig. S3.** A schematic diagram of the timeline of the experiments.

## Data Availability

The data supporting the findings of this study are available within the article.

## Abbreviations

miRNAs or miRs: MicroRNAs
BPD: Bronchopulmonary dysplasia
PlGF: Placental growth factor
STAT3: Signal transducer and activator of transcription 3
SPF: Specific pathogen-free
SD: Sprague–Dawley
NC: Negative control
ELISA: Enzyme-linked immunosorbent assay
IL: Interleukin
TNF-ɑ: Tumor necrosis factor-α
HRP: Horseradish peroxidase
H&E: Hematoxylin–eosin
PBS: Phosphate buffer solution
RIP: RNA binding protein immunoprecipitation
BALF: Bronchoalveolar lavage fluid
DAB: Diaminobenzidine
UTR: Untranslated region
WT: Wild type
MUT: Mutation
GAPDH: Glyceraldehyde-3-phosphate dehydrogenase
BCA: Bicinchoninic acid
ECL: Enhanced chemiluminescence
ANOVA: Analysis of variance

## References

[CR1] Ataie-Kachoie P, Pourgholami MH, Richardson DR, Morris DL (2014). Gene of the month: interleukin 6 (IL-6). J Clin Pathol.

[CR2] Bellik L, Vinci MC, Filippi S, Ledda F, Parenti A (2005). Intracellular pathways triggered by the selective FLT-1-agonist placental growth factor in vascular smooth muscle cells exposed to hypoxia. Br J Pharmacol.

[CR3] Chandrasekaran KS, Sathyanarayanan A, Karunagaran D (2017). miR-214 activates TP53 but suppresses the expression of RELA, CTNNB1, and STAT3 in human cervical and colorectal cancer cells. Cell Biochem Funct.

[CR4] Chao CM, van den Bruck R, Lork S, Merkle J, Krampen L, Weil PP (2018). Neonatal exposure to hyperoxia leads to persistent disturbances in pulmonary histone signatures associated with NOS3 and STAT3 in a mouse model. Clin Epigenet.

[CR5] Chen X, Du J, Jiang R, Li L (2018). MicroRNA-214 inhibits the proliferation and invasion of lung carcinoma cells by targeting JAK1. Am J Transl Res.

[CR6] Cheng X, Shen Y, Li R (2014). Targeting TNF: a therapeutic strategy for Alzheimer's disease. Drug Discov Today.

[CR7] Das P, Shah D, Bhandari V (2021). miR34a: a novel small molecule regulator with a big role in bronchopulmonary dysplasia. Am J Physiol Lung Cell Mol Physiol.

[CR8] Dong J, Carey WA, Abel S, Collura C, Jiang G, Tomaszek S (2012). MicroRNA-mRNA interactions in a murine model of hyperoxia-induced bronchopulmonary dysplasia. BMC Genomics.

[CR9] Doyle LW, Cheong JL, Ehrenkranz RA, Halliday HL. Early (< 8 days) systemic postnatal corticosteroids for prevention of bronchopulmonary dysplasia in preterm infants. Cochrane Database Syst Rev. 2017;10:CD001146 10.1002/14651858.CD001146.pub5 PMC6485683 29063585

[CR10] Dravet-Gounot P, Torchin H, Goffinet F, Aubelle MS, El Ayoubi M, Lefevre C (2018). Bronchopulmonary dysplasia in neonates born to mothers with preeclampsia: impact of small for gestational age. PLoS ONE.

[CR11] Du L, Borkowski R, Zhao Z, Ma X, Yu X, Xie XJ (2013). A high-throughput screen identifies miRNA inhibitors regulating lung cancer cell survival and response to paclitaxel. RNA Biol.

[CR12] Durrani-Kolarik S, Pool CA, Gray A, Heyob KM, Cismowski MJ, Pryhuber G (2017). miR-29b supplementation decreases expression of matrix proteins and improves alveolarization in mice exposed to maternal inflammation and neonatal hyperoxia. Am J Physiol Lung Cell Mol Physiol.

[CR13] Gonsalves CS, Li C, Mpollo MS, Pullarkat V, Malik P, Tahara SM (2015). Erythropoietin-mediated expression of placenta growth factor is regulated via activation of hypoxia-inducible factor-1alpha and post-transcriptionally by miR-214 in sickle cell disease. Biochem J.

[CR14] Isayama T, Lee SK, Yang J, Lee D, Daspal S, Dunn M (2017). Revisiting the definition of bronchopulmonary dysplasia: effect of changing panoply of respiratory support for preterm neonates. JAMA Pediatr.

[CR15] Janer J, Andersson S, Haglund C, Karikoski R, Lassus P (2008). Placental growth factor and vascular endothelial growth factor receptor-2 in human lung development. Pediatrics.

[CR16] Li QR, Tan SR, Yu J, Yang J (2018). MicroRNA-124 alleviates hyperoxia-induced inflammatory response in pulmonary epithelial cell by inhibiting TLR4/NF-kappaB/CCL2. Int J Clin Exp Pathol.

[CR17] Liao J, Lin J, Lin D, Zou C, Kurata J, Lin R (2017). Down-regulation of miR-214 reverses erlotinib resistance in non-small-cell lung cancer through up-regulating LHX6 expression. Sci Rep.

[CR18] Liu C, Luo J, Zhao YT, Wang ZY, Zhou J, Huang S (2018). TWIST1 upregulates miR-214 to promote epithelial-to-mesenchymal transition and metastasis in lung adenocarcinoma. Int J Mol Med.

[CR19] Newell LF, Holtan SG, Yates JE, Pereira L, Tyner JW, Burd I (2017). PlGF enhances TLR-dependent inflammatory responses in human mononuclear phagocytes. Am J Reprod Immunol.

[CR20] Ogawa R, Mori R, Iida K, Uchida Y, Oshiro M, Kageyama M (2017). Effects of the early administration of sivelestat sodium on bronchopulmonary dysplasia in infants: a retrospective cohort study. Early Hum Dev.

[CR21] Principi N, Di Pietro GM, Esposito S (2018). Bronchopulmonary dysplasia: clinical aspects and preventive and therapeutic strategies. J Transl Med.

[CR22] Qi L, Jiang J, Jin P, Kuang M, Wei Q, Shi F (2018). Expression patterns of claudin-5 and its related signals during luteal regression in pseudopregnant rats: the enhanced effect of additional PGF treatment. Acta Histochem.

[CR23] Rocha G, Proenca E, Guedes A, Carvalho C, Areias A, Ramos JP (2012). Cord blood levels of IL-6, IL-8 and IL-10 may be early predictors of bronchopulmonary dysplasia in preterm newborns small for gestational age. Dis Markers.

[CR24] Rovani MT, Ilha GF, Gasperin BG, Nobrega JE, Siddappa D, Glanzner WG (2017). Prostaglandin F2alpha-induced luteolysis involves activation of signal transducer and activator of transcription 3 and inhibition of AKT signaling in cattle. Mol Reprod Dev.

[CR25] Ruiz-Camp J, Quantius J, Lignelli E, Arndt PF, Palumbo F, Nardiello C (2019). Targeting miR-34a/Pdgfra interactions partially corrects alveologenesis in experimental bronchopulmonary dysplasia. EMBO Mol Med.

[CR26] Shah D, Sandhu K, Das P, Aghai ZH, Andersson S, Pryhuber G (2020). miR-184 mediates hyperoxia-induced injury by targeting cell death and angiogenesis signalling pathways in the developing lung. Eur Respir J.

[CR27] Sitia R, Rubartelli A (2018). The unconventional secretion of IL-1beta: handling a dangerous weapon to optimize inflammatory responses. Semin Cell Dev Biol.

[CR28] Stoll BJ, Hansen NI, Bell EF, Walsh MC, Carlo WA, Shankaran S (2015). Trends in care practices, morbidity, and mortality of extremely preterm neonates, 1993–2012. JAMA.

[CR29] Sun Y, Kuek V, Liu Y, Tickner J, Yuan Y, Chen L (2018). MiR-214 is an important regulator of the musculoskeletal metabolism and disease. J Cell Physiol.

[CR30] Syed M, Das P, Pawar A, Aghai ZH, Kaskinen A, Zhuang ZW (2017). Hyperoxia causes miR-34a-mediated injury via angiopoietin-1 in neonatal lungs. Nat Commun.

[CR31] Wang J, Bao L, Yu B, Liu Z, Han W, Deng C (2015). Interleukin-1 beta promotes epithelial-derived alveolar elastogenesis via alphavbeta6 integrin-dependent TGF-beta activation. Cell Physiol Biochem.

[CR32] Wang Y, Huang C, Chintagari NR, Xi D, Weng T, Liu L (2015). miR-124 regulates fetal pulmonary epithelial cell maturation. Am J Physiol Lung Cell Mol Physiol.

[CR33] Will JP, Hirani D, Thielen F, Klein F, Vohlen C, Dinger K (2019). Strain-dependent effects on lung structure, matrix remodeling, and Stat3/Smad2 signaling in C57BL/6N and C57BL/6J mice after neonatal hyperoxia. Am J Physiol Regul Integr Comp Physiol.

[CR34] Yang WC, Chen CY, Chou HC, Hsieh WS, Tsao PN (2015). Angiogenic factors in cord blood of preterm infants predicts subsequently developing bronchopulmonary dysplasia. Pediatr Neonatol.

[CR35] Yang Y, Li Z, Yuan H, Ji W, Wang K, Lu T (2019). Reciprocal regulatory mechanism between miR-214-3p and FGFR1 in FGFR1-amplified lung cancer. Oncogenesis.

[CR36] Yue Y, Luo Z, Liao Z, Zhang L, Liu S, Wang M (2019). Excessive activation of NMDA receptor inhibits the protective effect of endogenous bone marrow mesenchymal stem cells on promoting alveolarization in bronchopulmonary dysplasia. Am J Physiol Cell Physiol.

[CR37] Zhang ZQ, Huang XM, Lu H (2014). Early biomarkers as predictors for bronchopulmonary dysplasia in preterm infants: a systematic review. Eur J Pediatr.

[CR39] Zhang Z, Zhong Y, Li X, Huang X, Du L (2020). Anti-placental growth factor antibody ameliorates hyperoxia-mediated impairment of lung development in neonatal rats. Braz J Med Biol Res..

[CR38] Zhao Y, Ponnusamy M, Zhang L, Zhang Y, Liu C, Yu W (2017). The role of miR-214 in cardiovascular diseases. Eur J Pharmacol.

