# Correction to: MicroRNA‑214 promotes alveolarization in neonatal rat models of bronchopulmonary dysplasia via the PlGF‑dependent STAT3 pathway

**DOI:** 10.1186/s10020-021-00396-y

**Published:** 2021-10-21

**Authors:** Zhi‑Qun Zhang, Hui Hong, Jing Li, Xiao‑Xia Li, Xian‑Mei Huang

**Affiliations:** Department of Neonatology, Affiliated Hangzhou First People’s Hospital, Zhejiang University School of Medicine, No. 261, Huansha Road, Hangzhou, 310000 Zhejiang Province People’s Republic of China

## Correction to: Mol Med (2021) 27:109 https://doi.org/10.1186/s10020-021-00374-4

Following publication of the original article (Zhang et al. 2021), the authors informed us that they misused the wrong file of Fig. 7A. The correct Fig. 7 is given below.

The original article has been corrected.

**Fig. 7:**
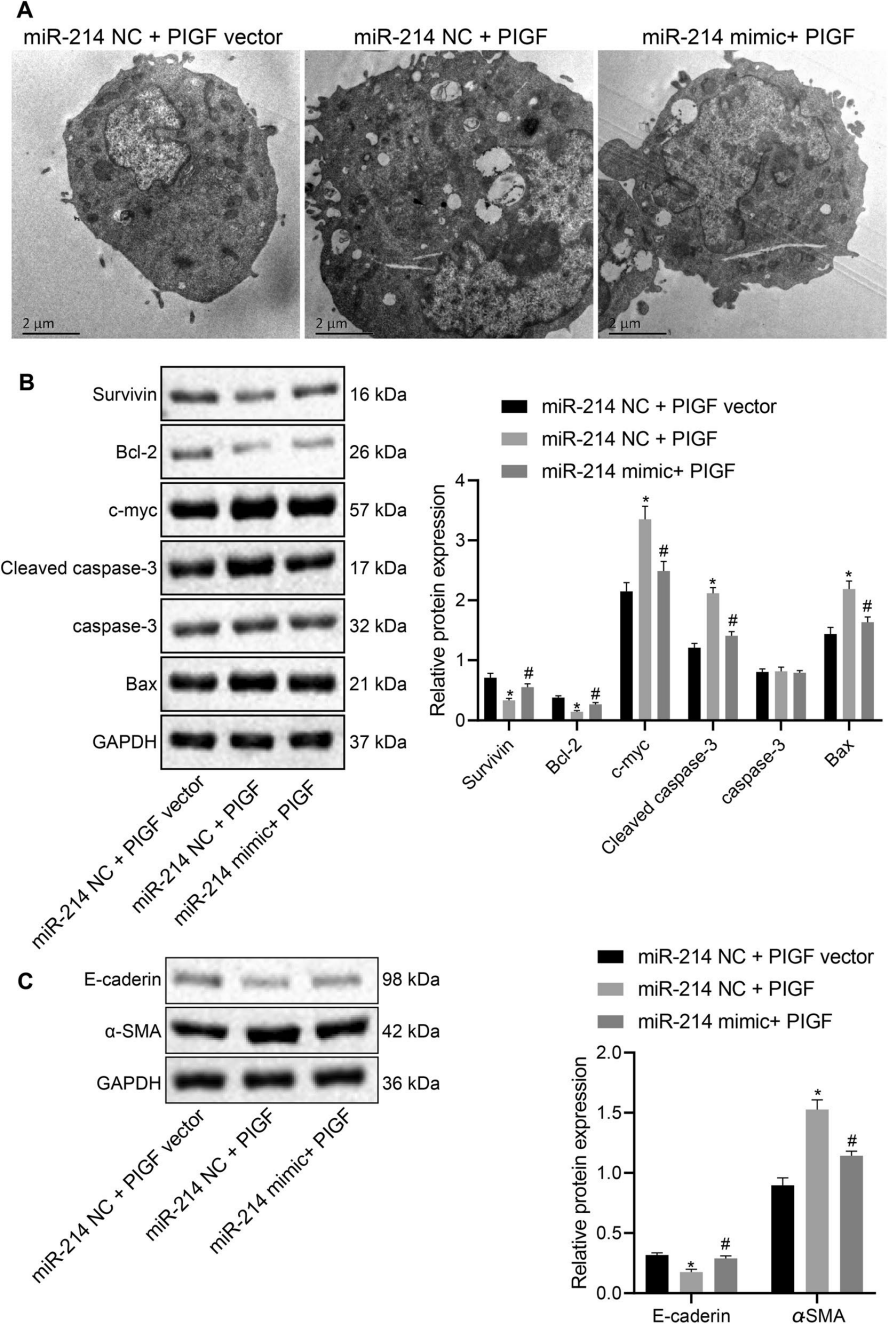
miR-214 overexpression blocks the effect of the activated STAT3 pathway on bronchial embryonic pulmonary epithelial cells by inhibiting PlGF. **A** The ultrastructure of alveolar epithelial cells under TEM (×10,000). **B** Western blot analysis to quantify the expression of antiapoptotic proteins (Survivin and Bcl-2) and proapoptotic proteins (Bax, c-myc, and cleaved caspase-3) proteins in embryonic pulmonary epithelial cells. **C** Western blot analysis to quantify the expression of the epithelial cell marker E-cadherin and the fibrosis marker α-SMA in embryonic pulmonary epithelial cells. Data are summarized as mean ± standard deviation. *p < 0.05 vs. pulmonary epithelial cells transfected with miR-214 NC and PlGF NC. ^#^p < 0.05 vs. pulmonary epithelial cells transfected with miR-214 NC and PlGF. Multiple comparisons were performed using one-way ANOVA, followed by Tukey’s post hoc test. Each experiment was repeated three times

## References

[CR1] Zhang Z-Q, Hong H, Li J, Li X-X, Huang X-M (2021). MicroRNA-214 promotes alveolarization in neonatal rat models of bronchopulmonary dysplasia via the PlGF-dependent STAT3 pathway. Mol Med.

