# Supplementary material for: MicroRNA-214 promotes alveolarization in neonatal rat models of bronchopulmonary dysplasia via the PlGF-dependent STAT3 pathway
Source: Mol Med. 2021 Sep 16;27:109. doi: 10.1186/s10020-021-00374-4 (PMC8444414; doi:10.1186/s10020-021-00374-4)
Supplement: Supplementary file 1 — Additional file 1: Table S1. Primer sequences of RT-qPCR [file 10020_2021_374_MOESM1_ESM.docx]

**Supplementary Table 1** Primer sequences of RT-qPCR

| Gene | Primer sequences (3’-5’) |
| --- | --- |
| rno-GAPDH | F: GCCATCAACGACCCCTTCAT |
| rno-GAPDH | R: AGATGGTGATGGGTTTCCCG |
| rno-U6 | F: AGTGCCTGCTTCGGCAGCACAT |
| rno-U6 | R: AAATATGGAACGCTTCACGAAT |
| rno-miR-214 | F: ACAGCAGGCACAGACAGGCAG |
| rno-miR-214 | R: CGACAGTTGCTATGCGATGCA |
| rno-PlGF | F: GGGCACTTGCTCTGCTTTTG |
| rno-PlGF | R: GCATCTGACAACTCGGGCTA |

Note: GAPDH, glyceraldehyde-3-phosphate dehydrogenase; PlGF, placental growth factor; eNOS, endothelial nitric-oxide synthase; F, forward; R, reverse.
